# Pulmonary Embolism Risk Assessment Using Blood Copeptin Concentration and Pulmonary Arteries Thrombotic Burden Evaluated by Computer Tomography

**DOI:** 10.3390/jpm12122084

**Published:** 2022-12-19

**Authors:** Mihai Ștefan Cristian Haba, Ionut Tudorancea, Radu Ștefan Miftode, Irene Paula Popa, Ovidiu Mitu, Cosmin Teodor Mihai, Raluca Maria Haba, Viviana Aursulesei Onofrei, Antoniu Octavian Petris, Irina Iuliana Costache, Danisia Haba, Laurentiu Șorodoc

**Affiliations:** 1 Department of Internal Medicine I, Faculty of Medicine, University of Medicine and Pharmacy “Grigore T. Popa”, 700115 Iasi, Romania; 2 Department of Morpho-Functional Sciences II-Physiology, Faculty of Medicine, University of Medicine and Pharmacy “Grigore T. Popa”, 700115 Iasi, Romania; 3 Advanced Research and Development Center for Experimental Medicine (CEMEX), “Grigore T. Popa” University of Medicine and Pharmacy of Iasi, 700115 Iasi, Romania; 4 Faculty of General Medicine, Grigore T. Popa University of Medicine and Pharmacy, 700115 Iași, Romania; 5 Department of Oral and Maxillofacial Surgery, Faculty of Dental Medicine, “Gr. T. Popa” University of Medicine and Pharmacy, 16 Universitatii Str., 700115 Iasi, Romania; 6 Department of Internal Medicine III, Faculty of Medicine, University of Medicine and Pharmacy “Grigore T. Popa”, 700115 Iasi, Romania

**Keywords:** pulmonary embolism, copeptin, Mastora score

## Abstract

(1) Background: Pulmonary embolism (PE) represents the third most important cardiovascular cause of death after myocardial infarction and stroke. The proper management of this condition is dependent on adequate risk stratification, due to the life-threatening complications of more aggressive therapies such as thrombolysis. Copeptin is a surrogate marker of vasopressin which is found increased in several cardiovascular conditions. The Mastora score is an imagistic evaluation of the degree of pulmonary arteries thrombotic burden based on computed tomography angiography. In this study, we aimed to evaluate the diagnostic and prognostic role of copeptin in patients with acute PE. Furthermore, we analyzed the relationship between copeptin and Mastora score and their role in PE risk profiling. (2) Methods: We conducted a single center prospective study that included 112 patients with PE and 53 healthy volunteers. Clinical and paraclinical parameters, together with plasma levels of copeptin and the Mastora score, were evaluated in all patients after admission. (3) Results: Copeptin levels were significantly increased in PE patients compared with the general population (26.05 vs. 9.5 pmol/L, *p* < 0.001), while receiver operating characteristic (ROC) analysis revealed an AUC of 0.800 (95% CI 0.728–0.873, *p* < 0.001). Copeptin directly correlated with the Mastora score (r = 0.535, *p* = 0.011) and both parameters were strong predictors for adverse clinical events and death. Receiver operating characteristic (ROC) analysis for death within 30 days revealed a copeptin cut-off of 38.36 pmol/L, which presented a specificity of 79.6% and a sensitivity of 88.9%, and a Mastora score cut-off of 82 points, which presented a specificity of 74.8% and a sensitivity of 77.8%. (4) Conclusions: Our results showed that copeptin and the Mastora score are both correlated with adverse cardiovascular events and mortality in PE patients, and this may pave the way for their use in clinical practice, helping physicians to select the best therapeutical management.

## 1. Introduction

With a worldwide incidence of 39–115 cases per 100,000 people and a mortality rate up to 30%, pulmonary embolism (PE) represents the third most important cause of cardiovascular death after myocardial infarction and stroke [1]. In the absence of accurate diagnosis strategies, PE can easily be misdiagnosed as its symptomatology is polymorphic, varying from mild symptoms such as thoracic pain to severe presentations such as acute respiratory failure or cardiac arrest. Intriguingly, registry data have revealed that over 59% of cases of PE resulting in death are diagnosed postmortem [2]. Even after adequate diagnosis, PE patients admitted with mild or moderate symptoms can present with a poor clinical evolution leading to death. Treatment of PE can vary significantly from oral and parenteral anticoagulants to more radical therapies such as systemic thrombolysis, catheter guided thrombolysis, interventional mechanical thrombus removal and surgical thrombectomy [1,3,4]. Thrombolysis, interventional and surgical procedures are usually reserved for patients with hemodynamic instability, due to the increased risk of life-threatening adverse effects. However, a subgroup of patients with normal blood pressure may have a short-term mortality rate of up to 24.5% [5] and thus a more aggressive therapeutic approach is indicated in such situations. Therefore, finding new approaches to improve the accuracy of PE risk profiling and prognosis is mandatory to be able to choose the best therapeutic management.

The main pathophysiological process of PE is determined by the thrombotic burden which leads to increased pulmonary artery pressure and subsequent acute right ventricle (RV) dysfunction [6]. Current strategies for predicting PE risk are based on clinical, imagistic and biomarker parameters which analyze clot presence and burden as well as RV dysfunction. Parameters such as Pulmonary Embolism Severity Index (PESI) and its simplified version (sPESI), thrombosis and cardiovascular biomarkers (NT-proBNP, Troponin, D-dimers) and echocardiographic markers of RV dysfunction (right ventricular diameter, tricuspid annular plane systolic elevation, systolic pulmonary artery pressure) have been presented in various studies and guidelines; however, none of them were able to specifically predict PE-related death [7,8,9].

Thrombotic burden can be directly evaluated with computed tomography pulmonary angiography (CTPA) and imagistic derived obstruction indexes such as the Mastora score can be calculated. The Mastora score is used to establish the degree of obstruction of the pulmonary, lobar, segmental and subsegmental arteries [10]. Even though this score has shown promising correlations with PE severity, it has not yet been implemented into everyday clinical practice [11,12].

Although troponin and NT-proBNP are the most used biomarkers for the diagnosis and prognosis of PE, there is continuous interest in the discovery of novel biomarkers to increase the accuracy of PE risk profiling. One such biomarker is vasopressin, which is usually expressed in the context of the pathophysiological stress induced by cardiopulmonary conditions and has an important role in promoting the process of cardiac remodeling. However, determination of circulating vasopressin is technically challenging and unfeasible in emergency scenarios; blood copeptin (the C-terminal portion of vasopressin), on the other hand, may be used as a surrogate marker for the activity of vasopressin [13]. High copeptin levels have been found in myocardial infarction, heart failure and several studies have shown that it may also play a role in PE [14,15,16,17,18].

In this study we tested the hypothesis that high copeptin levels and high thrombotic burden evaluated through the Mastora score could be used as predictors for a worse outcome of pulmonary embolism. Therefore, we tested if copeptin and the Mastora score are associated with the occurrence of adverse events during hospitalization and with 30-day mortality.

## 2. Materials and Methods

### 2.1. Study Design and Population

We conducted a prospective case-control study that evaluated 112 consecutively enrolled patients with acute pulmonary embolism diagnosed by CTPA and admitted in the Cardiology Clinic of the St. Spiridon Emergency County Hospital (Iași, Romania) between June 2021 and July 2022. The control group included 53 sex- and age-matched volunteers who were admitted to our outpatient clinics. The exclusion criteria for the patients included in both groups were as follows: acute left ventricular heart failure, acute coronary syndrome, chronic pulmonary hypertension, severe chronic obstructive lung disease, end-stage renal failure, sepsis, acute cerebrovascular disease and acute or chronic aortic dissection. To obtain a comprehensive medical history, detailed anamnesis was performed and patients’ personal and hospital medical files were reviewed. The clinical endpoints of the study were death within 30 days of admission and the occurrence of adverse events (hemodynamic instability, the use of vasopressor drugs, admission to ICU, thrombolysis) during hospitalization.

After admission, a venous blood sample was collected from all patients and was centrifuged at 3000 rpm for 15 min to separate plasma. Copeptin levels were measured using a Copeptin (CPP) ELISA kit (Antibodies online GmbH, Aachen, Germany), with a detection range between 78–5000 pg/mL, with a minimum detection limit of 78 pg/mL and a sensitivity of 19.5 pg/mL. Echocardiography was performed using a General Electric Vivid^TM^ V7 ultrasound device (General Electric, Boston, CA, USA) to evaluate PE specific ultrasound parameters such as right ventricular diameter (RVd), tricuspid annular plane systolic elevation (TAPSE) and estimated systolic pulmonary arterial pressure (sPAP). Based on clinical and paraclinical parameters, a pulmonary embolism severity index (PESI) score was determined for PE patients. Furthermore, patients were classified according to the European Society of Cardiology (ESC) guidelines algorithm into low, intermediate-low, intermediate-high and high risk [1].

Patients’ chest CTPA was performed with a 64 slices Incisive^TM^ CT scanner (Philips, Amsterdam, The Netherlands) using Ultravist 370^TM^ (Bayer, Berlin, Germany) intravenous contrast. The collected CT images were interpreted by two radiologists in a randomized order, which evaluated RV/LV ration and the clotting burden using Mastora scoring, according to the algorithm previously presented by Mastora et al. [10]. The clot burden was analyzed at the level of five mediastinal arteries (pulmonary artery trunk, right and left main pulmonary arteries, right and left interlobar arteries), six lobar arteries (right truncus anterior, right middle lobe pulmonary artery, right lower lobe pulmonary artery, left upper lobe pulmonary artery, middle lobe pulmonary artery and the left upper lobe pulmonary artery) and 20 segmental pulmonary arteries (three right and left upper lobe segmental arteries, the two right middle lobe and left upper lobe segmental arteries, and the five right and left lower lobe segmental arteries). The obstructed surface of each artery was assessed based on a 5-point scale estimate (1: <25%; 2: 25–49%; 3: 50–74%; 4: 75–99%; 5: 100%). The final obstruction score was represented by the summation of the percentage of obstruction for each of the 31 evaluated arteries.

The study protocol was approved by the Ethics Committee of the Grigore T. Popa University of Medicine and Pharmacy and by the Ethics Committee of the St. Spiridon Emergency Clinical Hospital. All research was conducted according to the ethical guidelines of the Declaration of Helsinki Principles, as revised in 2013. All patients signed a standard written informed consent to participate in this study.

### 2.2. Statistical Analysis

The data gathered in our study were analyzed using IBM SPSS Statistics for Windows v.26.0 (IBM, Armonk, NY, USA). Normal distribution of the continuous variables was assessed using the Kolmogorov–Smirnov test. In this paper, normally distributed continuous variables are reported as mean ± standard deviation (SD), together with the minimum and maximum value. Continuous variables not normally distributed were reported as medians with interquartile ranges (IQRs). Categorical variables are expressed as frequencies and percentages. Parametric (independent sample t-test) and non-parametric (Mann–Whitney U) tests were used to compare data from the study and control groups. The Pearson coefficient was used to measure correlation between continuous variables, while the Spearman correlation coefficient was used for non-parametric variables. We used binary logistic regression to estimate the influence of continuous predictors on binary outcomes. The Hosmer–Lemershow test was performed to assess the quality of the logistic regression model. Linear regression was conducted to observe how variables vary between each other. Receiver operating characteristic (ROC) analysis was used to determine the diagnostic properties of copeptin and the prognostic properties of both copeptin and the Mastora score in relation to adverse events and death. ROC analysis was also used to identify a cut-off value for the same variables. A *p*-value < 0.05 was considered statistically significant.

## 3. Results

### 3.1. General Charactersistics

A total of 165 patients were included in our study, out of which 112 patients diagnosed with acute PE represented the study group, while 53 volunteers represented the control group. Both study and control groups presented similar demographic distribution for age and sex. A comparison of the baseline characteristics of patients in the study group and the control group revealed no significant statistical differences regarding the prevalence of PE risk factors: active cancer, history of recent surgery, arterial hypertension and diabetes mellitus. Analysis of the clinical and paraclinical parameters of both groups showed a significant statistical difference in markers specific for PE (heart rate, systolic blood pressure, oxygen saturation in ambient air, leukocytes, C-reactive protein, D-dimers). The general characteristics of the patients included in the study are presented in Table 1.

In the study group, 30 (26.9%) of the patients presented low-risk PE, 49 (41.9%) presented intermediate-low-risk PE, 20 (17.1%) presented intermediate-high-risk PE and 13 (11.1%) presented high-risk PE. There was a significant statistical difference regarding age according to patients’ risk level, as low-risk PE patients were younger than the other groups of risk (*p* < 0.001). Statistical difference was also observed in clinical parameters of these patients such as heart rate, blood pressure and oxygen saturation in ambient air (*p* < 0.001). However, there was no statistical difference between the incidence of symptoms and the average Wells and Geneva score between risk groups. In our study group, the number of adverse cardiac events was 26 (23.2%), while 9 (8%) patients died within 30 days from admission. As expected, patients included in the low-intermediate-, high-intermediate- and high-risk groups presented a significantly increased number of adverse cardiac events and death (*p* < 0.001). Demographic and general characteristics according to risk group are summarized in Table 2.

**Figure 1:**
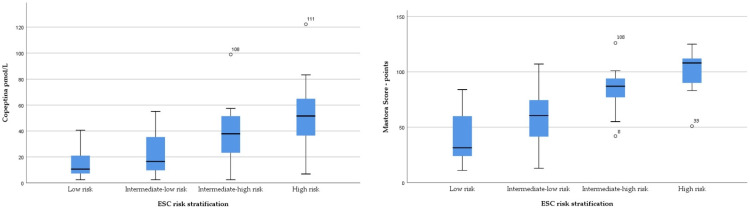
Copeptin and Mastora score distributions according to ESC risk stratification.

A subgroup analysis of the PE patients, according to risk groups, revealed a significant statistical difference in the biological and imagistic parameters specific to PE, such as hsTnI, NT-proBNP, echographic RVd, TAPSE and sPAP (*p* < 0.001). However, D-dimer levels were similar in PE patients between all four risk groups (*p* = 0.154). There was a significant increase (*p* < 0.001) in copeptin expression from low-risk patients (9.86 pmol/L, IQR 6.89–17.30) to low-intermediate-risk patients (18.63 pmol/L, IQR 9.31–35.76), high-intermediate-risk patients (37.89 pmol/L, IQR 25.04–51.01) and up to high-risk patients (51.45 pmol/L, IQR 36.5–75.09). Likewise, there was a significant increase in Mastora score from low-risk patients (41.93 ± 21.03 points) to low-intermediate-risk patients (59.63 ± 24.05 points), to high-intermediate-risk patients (83.57 ± 17.34 points) and up to high-risk patients (101.23 ± 20.62 points). The biological and imagistic parameters for the PE groups are shown in Table 3. Copeptin and Mastora score distributions according to risk group are shown in Figure 1.

### 3.2. Copeptin for Diagnosis of PE

**Figure 2:**
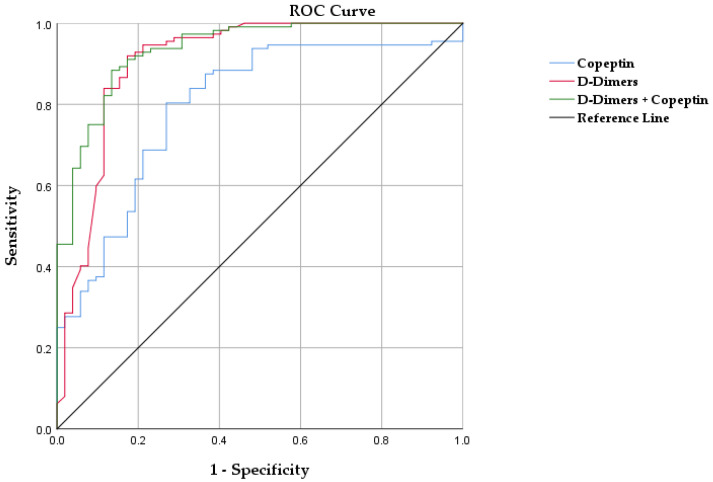
ROC curve analysis of copeptin and D-dimers for PE diagnosis.

As copeptin expression was statistically significantly higher in the PE patients’ group than in the control group, we aimed to evaluate its diagnostic role compared to the currently used biomarker, D-dimers. Consequently, we performed an ROC analysis, which revealed an AUC of 0.800 (95% CI 0.728–0.873, *p* < 0.001), significantly smaller than the AUC of D-dimers of 0.908 (95% CI 0.851–0.965, *p* < 0.001). When evaluating the combined predictive value of both D-dimers and copeptin, they presented an AUC of 0.935 (*p* < 0.001, CI 95% 0.896–0.974). Analyzing the ROC curve for the diagnosis of PE, we found a cut-off value for copeptin of 7.96 pmol/L, with a sensitivity of 80.4% and a specificity of 73.6%, while the cut-off for D-dimers was 3.55 µg/mL with a sensitivity of 81.3% and a specificity of 88.7%. ROC curves for both copeptin and D-dimers are illustrated in Figure 2.

### 3.3. Copeptin and Mastora Score for Evaluation of PE Prognosis

Both copeptin and the Mastora score showed statistically significant differences in values corresponding to the classification of PE patients according to the ESC risk stratification. Furthermore, in our study, copeptin and the Mastora score also presented a significant correlation with clinical, biologic and imagistic parameters that were already validated as predictors for the severity of PE, such as SPB, HR, NT-proBNP, Troponin, RVd, TAPSE, sPAP, RV/LV ratio and PESI score, as well as with adverse cardiac events and death. Correlations between copeptin, the Mastora score and clinical and paraclinical parameters are summarized in Table 4.

**Figure 3:**
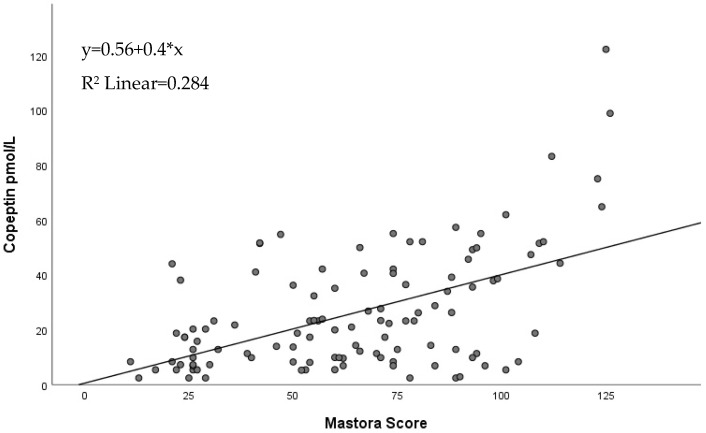
Linear regression between Mastora score and copeptin.

Regression analysis revealed a linear relationship between the Mastora score and copeptin characterized by the following equation: y = 0.56 + 0.4 * x, where y represents the copeptin value and x represent the value of the Mastora score, which was applicable for 28.4% of the PE patients, as seen in Figure 3.

**Figure 4:**
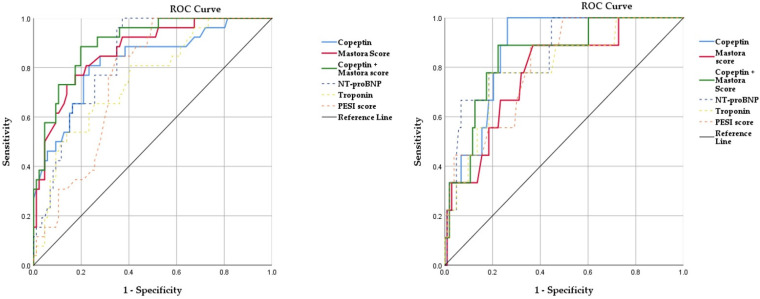
ROC curves for prediction of adverse events (**left**) and death (**right**).

Further ROC evaluation of the Mastora score, copeptin, NT-proBNP, hsTnI and PESI score, showed that Mastora was the best predictor for adverse events (AUC 0.871, *p* < 0.001, CI 95% 0.796–0.945). When assessing only biomarkers alone, NT-proBNP presented slightly better prediction properties than copeptin (AUC 0.853 vs. 0.820), while hsTnI had poorer risk stratification values (AUC 0.736). Interestingly, when performing the same analysis for death within 30 days as an end-point, the best predictor was copeptin (AUC 0.883, *p* < 0.001, CI 95% 0.809–0.957), followed by NT-proBNP, PESI score, Mastora score and, finally, hsTnI. When evaluating the combined probability of copeptin and the Mastora score, it performed better in determining the occurrence of adverse events (AUC 0.902, *p* < 0.001, CI 95% 0.841–0.962), but did not outperform copeptin or NT-proBNP in predicting mortality (AUC 0.85, *p* = 0.001, CI 95% 0.724–0.967). Analysis of the coordinate points on the ROC curve showed a copeptin cut-off value for adverse events of 26.57 pmol/L, with a sensitivity of 80.2% and a specificity of 76.7%, and a Mastora score cut-off value for adverse events of 78 points, with a sensitivity of 80.8% and a specificity of 79.1%. A similar evaluation revealed a copeptin cut-off value for death of 38.36 pmol/L, with a sensitivity of 88.9% and a specificity of 79.6%, and a Mastora score cut-off value of 82 points, with a sensitivity of 77.8% and a specificity of 74.8%. The ROC curve analysis data for Mastora score and copeptin in relation to adverse events and death are presented in Table 5 and Figure 4.

Additionally, binary logistic regression assessment revealed that a one unit increase in copeptin resulted in a 4.9% higher probability of adverse events and a 4.8% higher probability of death. Similarly, a one point increase in the Mastora score led to a 6.5% higher probability of adverse events. However, the Mastora score increase did not show a statistically significant effect on short-term mortality. Data from binary logistic regression are shown in Table 6.

## 4. Discussion

Acute PE represents a severe condition with polymorphic symptomatology where timely diagnosis can be challenging. Moreover, as PE is associated with important mortality rates, a precise assessment of the risk of adverse events is mandatory to select the best therapeutic management for these patients. Besides clinical examination, biomarkers together with imagistic evaluation (echocardiography, CTPA) form the toolkit which facilitates physicians being able to determine the diagnosis of acute PE and to evaluate its prognosis [1]. In this study, we tested the hypothesis that the expression of copeptin is high in patients with PE when compared to the general population. Additionally, we tested the premise that the association between copeptin and the Mastora score may be used for risk stratification and short-term prognosis of PE patients. We evaluated copeptin expression in patients diagnosed with acute PE and compared its pretest diagnostic capabilities with the currently used biomarkers, D-dimers. Subsequently, after calculating the clot burden through the CTPA Mastora score, we evaluated whether this parameter, together with copeptin, can better predict the prognosis of PE patients.

In our research, plasma levels of copeptin were significantly higher in acute PE patients when compared with a control group of patients from the general population having the same demographic profile and similar risk factors. Additionally, ROC curve analysis revealed a cut-off value for copeptin of 7.96 pmol/L with a sensitivity of 80.4% and a specificity of 73.6%. Our results are in concordance with previously published papers where copeptin presented with a 68.1–71.9% sensitivity and a 83.7–85% specificity for acute PE [14,19]. When compared with D-dimers, the diagnostic power of copeptin is slightly lower, presenting with an AUC of 0.800 (95% CI 0.728–0.873) vs. the AUC of 0.908 (95% CI 0.851–0.965, *p* < 0.001) for D-dimers. Currently used medical practice guidelines recommend the use of D-dimers as the main biomarker for pretest probability before CTPA [1]. In our study, D-dimers presented a sensitivity of 81.3% and a specificity of 88.7%. Nevertheless, similar studies evaluating D-dimers for PE diagnosis presented comparable or better sensitivity values up to 96%, while specificity varied considerably from 41 up to 70% [20,21]. Furthermore, D-dimer levels are increased in certain categories of patients where PE probability is already high, such as pregnancy or cancer, clinical scenarios in which a pretest diagnosis based on this biomarker becomes unreliable [22,23]. In these situations, the addition of a supplementary biomarker such as copeptin may be used to increase the pretest evaluation accuracy. In our study, there was no direct statistical correlation between copeptin levels and cancer, results which are in accordance with previous published papers [24]. The literature shows no correlations between copeptin levels and uncomplicated pregnancy [25], though high copeptin blood levels can be found in complications such as pregnancy-induced hypertension and preeclampsia [26,27]. On the other hand, copeptin serum and plasma levels can be increased in renal pathology, sepsis or other cardiovascular conditions due to its role in the neurohormonal activation pathways [28,29,30]. Furthermore, in our study, the analysis of the combined probability of D-dimers and copeptin for PE diagnosis revealed an AUC of 0.935, which was higher than that of the individual biomarkers. According to our results, copeptin alone is not an ideal biomarker for acute PE diagnosis, especially when compared with the current standard of D-dimers. However, determining copeptin levels could become a complementary test which could enhance diagnostic probability in situations in which D-dimers determination offers inconclusive results.

The acute PE population in our study presented with a risk profile and mortality which was comparable to that in previously reported papers [31], thus enabling us to examine the role of copeptin and the Mastora score for prognostic assessment of PE. In the present research, copeptin levels were statistically significantly higher in each risk category of PE patients, starting with low risk and up to high risk. Furthermore, copeptin was correlated with biomarkers which already have been established as predictors for severe PE, such as troponin and NT-proBNP [7,8,32,33]. In PE patients, an increase in troponin or NT-proBNP levels is a reflection of right ventricular dysfunction and injury resulting from the acute rise in pressure in the pulmonary circulatory system [7]. Right ventricular dysfunction is the primary cause for hemodynamic imbalance in PE, and its presence is a critical determinant factor for clinical severity and prognosis for these patients [34]. In our study, besides the correlation with cardiac biomarkers, copeptin also correlated with RV dysfunction evaluated through echocardiography (RVd, sPAP, TAPSE) and CTPA (RV/LV ratio>1). Multiple studies have also validated these imagistic parameters as predictors for the clinical outcome of PE patients [9,35]. Our results are in accord with previous papers where copeptin was correlated with both paraclinical and imagistic markers of PE severity [14,19,31].

Even though RV dysfunction markers are primarily used for the risk stratification of PE patients, the localization and the extension of the thrombus are important factors that influence the pathological hemodynamic processes that occur in this condition. Besides diagnosis, CTPA can be used to offer a precise description of the obstruction at each level of the pulmonary circulation which could be used to predict the speed at which the disease may evolve. Consequently, Mastora et al. proposed a score assessing the degree of obstruction at the level of mediastinal, lobar and segmental pulmonary arteries [10]. In our study group, the Mastora score correlated with clinical markers (SBP), paraclinical biomarkers (NT-proBNP, D-dimers) and echocardiographic biomarkers for RV dysfunction (TAPSE, RVd). However, there was no statistically significant correlation between the Mastora score and troponin or sPAP. Furthermore, logistic regression revealed a statistically significant linear relationship between an increase in copeptin value and the Mastora score (y = 0.56 + 0.4 * x, R^2^ Linear = 0.284). These results are similar to previously reported results where the Mastora score was correlated with SBP, RV dysfunction and D-dimers [11,36,37]. Even though the relationship between the Mastora score and copeptin has not yet been evaluated in previous studies, our promising results highlight the importance of both these parameters in the pathophysiology of PE.

To better assess the risk stratification properties of both copeptin and the Mastora score, we used receiver operator curve (ROC) analysis to investigate the prediction role of these parameters together with NT-proBNP, troponin and PESI score for adverse events (hemodynamic instability, positive inotropic treatment, admission to ICU, thrombolysis) and short-term mortality. Regarding adverse events, the Mastora (AUC 0.871) score outperformed NT-proBNP, copeptin, PESI score and troponin. Moreover, the combined probability of the Mastora score and high copeptin levels presented the best AUC (0.902) for prediction of adverse cardiac events. Furthermore, our analysis revealed a Mastora score cut-off value for adverse events of 78 points, with a sensitivity of 80.8% and a specificity of 79.1%, and a copeptin cut-off value of 26.57 pmol/L, with a similar sensitivity of 80.2% and a specificity of 76.7%. When analyzing the ROC curves for mortality, copeptin outperformed the other parameters (AUC 0.883). The cut-off value for copeptin was 38.36 pmol/L, with a sensitivity of 88.9% and a specificity of 79.6%, and the Mastora score cut-off value was 82 points, presenting a lower sensitivity of 77.8% and a specificity of 74.8%. Binary logistic regression of the same parameters validated the role of the Mastora score and copeptin which could both predict an increase of adverse events. However, the odds ratio resulting from our analysis reflected only a small but statistically significant increase in death (4.8%), while a Mastora score increase was not associated with an increased odds ratio for mortality. On the other hand, the good correlations with other prognostic clinical and paraclinical parameters, together with the results from the ROC analysis, suggest that both copeptin and the Mastora score are important factors in risk profiling for PE patients. Our results complement previously reported data in which copeptin plays an important role in risk stratification for PE. In a study conducted on 107 patients with acute PE, Wyzgal et al. reported a cut-off value of 17.95 pmol/L with a sensitivity of 100% and a specificity of 49.5% for complicated clinical course [38]. Interestingly, in two studies analyzing over 800 patients, Hellenkamp et al. reported a copeptin cut-off value which was very close to our results, of 24 pmol/L, which can be used to stratify normotensive PE patients into intermediate-low risk and intermediate-high risk, and which can predict a 7.6 increase of PE-related death [18,39]. Regarding the Mastora score’s prognostic role, the literature’s data are more controversial. Račkauskienė et al. evaluated 106 patients with newly diagnosed PE and used the Mastora score to divide patients into “non-massive PE” and “massive PE” groups. According to their results, the “massive PE” patients were associated with a higher percentage of right ventricular dysfunction (86% vs. 50%) and a worse clinical outcome [11]. Similarly, after analyzing CTPA images from 131 PE patients, Venkatesh et al. reported that the Mastora score was significantly increased in patients who died within 30 days, and an increased Mastora score at the level of the mediastinal arteries (53% obstruction) presented with a sensitivity of 100% and a specificity of 76.5% for death [40]. On the other hand, Lerche et al. in research conducted on 246 patients reported no correlation between the Mastora score and biological parameters such as troponin, NT-proBNP or the severity of pulmonary embolism [41]. Likewise, Apfaltrer et al. described similar results, where the Mastora score was correlated with RV dysfunction but not with adverse clinical outcome [12]. Nevertheless, our research showed that the Mastora score correlated with cardiac biomarkers, imagistic markers of RV failure, and was a predictor for adverse events and death.

The data from our study showed that both copeptin and the Mastora score were directly correlated and were associated with adverse events and short-term mortality. These findings further emphasize the important pathophysiological relationship between thrombotic burden and the cardiovascular complications in PE patients. Furthermore, these promising results may suggest that both copeptin and the Mastora score could become valuable parameters in everyday medical practice.

### Limitations of the Study

The main limitations of our research derive from the fact that it was a single center study with a relatively small sample size. Furthermore, the exclusion criteria were broader to diminish the contributing effect of comorbidities on the deaths of PE patients, and therefore the results are more difficult to extrapolate. Nevertheless, the risk profile distribution as well as mortality rates were similar to those in previous reported studies. Another limitation is that copeptin was determined only immediately after diagnosis and hospital admission. Recurrent determinations during treatment, similar to other biomarkers, might offer even more insight into PE prognosis. However, to the best of our knowledge, this is the first study which aims to evaluate the relationship between copeptin and the thrombotic burden at the level of the pulmonary arteries.

## 5. Conclusions

Our study revealed that blood copeptin levels are significantly higher in PE patients when compared with the general population. Copeptin levels of 7.96 pmol/L presented with a sensitivity of 80.4% and a specificity of 73.6% for PE diagnosis in this study cohort. Even though D-dimers presented better diagnostic capabilities, copeptin may represent a supplementary pretest biomarker in special subgroups of PE patients where D-dimer determination alone can lead to false positive results.

More importantly, copeptin levels were directly correlated with the thrombotic burden at the level of the pulmonary arteries as evaluated by the Mastora score. Both parameters were significantly higher according to PE risk category (low risk, intermediate-low risk, intermediate-high risk and high risk) and were correlated with similar clinical, biological and imagistic risk markers such as SBP, troponin, NT-proBNP, RVd and TAPSE. Furthermore, our analysis revealed a copeptin cut-off for short-term mortality of 38.36 pmol/L, with a sensitivity of 88.9% and a specificity of 79.6%, and a Mastora score cut-off value of 82 points, presenting a sensitivity of 77.8% and a specificity of 74.8%

These promising results show that both copeptin and the Mastora score could become important elements in the risk profiling of patients with PE, helping physicians to select the best therapeutical approach.

## Figures and Tables

**Table 1:** General characteristics of the study groups.

Characteristics	Total (n = 165)				Pulmonary Embolism (n = 112)				Control Group (n = 53)				*p*-Value
	Min	Mean ± SD		Max	Min	Mean ± SD		Max	Min	Mean ± SD		Max	
Age (years)	18	62.04 ± 14.05		91	18	63.1 ± 14.10		91	30	59.6 ± 13.71		91	0.13
Gender (N, %)	Male 90 (54.5%)		Female 75 (45.4%)		Male 62 (55.35%)		Female 50 (44.6%)		Male 28 (52.8%)		Female 25 (47.2%)		0.566
Systolic blood pressure (mmHg)	50	127.45 ± 21.53		200	50	123.09 ± 21.90		200	110	136.66 ± 16.51		185	*<0.001*
Diastolic blood pressure (mmHg)	20	74.05 ± 12.98		110	20	75.12 ± 13.80		91	60	71.79 ± 10.83		110	0.12
Heart rate (bpm)	52	88.57 ± 19.22.02		155	52	94.07 ± 23.13		155	50	76.94 ± 13.53		111	*<0.001*
Oxygen saturation in ambient air (%)	65	94.21 ± 5.38		100	65	91.70± 4.81		99	97	99.45 ± 0.72		100	*<0.001*
Surgery within 30 days		12 (7.27%)				8 (7.14 %)				4 (7.54%)			0.328
Active cancer		15 (9.09%)				13 (11.6%)				2 (3.77%)			0.102
Post-partum		2 (1.21%)				1 (0.89%)				1 (1.88%)			0.331
Diabetes mellitus		32 (19.4%)				19 (16.9%)				13 (24.5%)			0.365
Arterial hypertension		81 (49.09%)				59 (52.7%)				22 (41.5%)			0.156
Smoking		52 (31.5%)				39 (34.8%)				13 (24.5%)			0.172
BMI (kg/m^2^)	17.15	26.10 ± 3.75		40.41	17.15	26.30 ± 4.15		40.41	20.32	25.66 ± 2.70		36.45	0.303
Leucocytes (×10^9^/L)	4.06	9.97 ± 3.63		23.9	4.06	10.51 ± 4.13		23.9	5.11	8.86 ± 1.79		15.33	*0.006*
Hemoglobin (g/L)	9.2	13.29 ± 1.58		17.9	9.2	13.17 ± 1.77		17.9	9.2	13.48 ± 1.19		17	0.249
Thrombocytes (×10^9^/L)	93	253.51 ± 100.20		745	96	253.45 ± 120.16		745	93	253.61 ± 54.55		415	0.992
CRP (mg/dL)	0.02	5.53 ± 11.51		115	0.08	7.64 ± 13.44		115	0.02	1.09 ± 1.77		12	*0.001*
Glucose (mg/dL)	70	117.90 ± 41.59		310	75	122.92 ± 47.84		310	70	107.47 ± 20.21		160	0.09
Creatinine (mg/dL)	0.42	0.97 ± 0.38		3.71	0.42	0.97 ± 0.43		3.71	0.6	0.98 ± 0.27		1.77	0.715
LVEF (%)	15	51.28 ± 7.43		65	15	51.4 ± 7.3		65	35	51.09 ± 0.98		65	0.809
	**Median (IQR)**				**Median (IQR)**				**Median (IQR)**				
D-dimer (µg/mL)	4.6 (1.72–5.21)				4.75 (4.32–5.56)				1.55 (0.5–2.1)				*<0.001*
NT-proBNP	86.3 (31.95–137.5)				1376 (556–3384)				15 (4.34–53)				*<0.001*
hsTnI	6.08 (1.38–12.13)				29 (8.98–96)				662 (100.5–2344.5)				*<0.001*
Copeptin (pmol/L)	12.82 (5.41–30.97)				26.05 (8.69–40.23)				9.50 (3.92–10.63)				*<0.001*

BMI—body mass index, SBP—systolic blood pressure, HR—heart rate, CRP—C-reactive protein, LVEF—left ventricle ejection fraction, hsTnI—high sensitive troponin, NT-proBNP—N-terminal pro b-type natriuretic peptide, SD—standard deviation. Italic style was used for statistically significant results.

**Table 2:** Demographic and general characteristics according to risk group.

PE Group (No, %)	Low-Risk PE 30 (26.8%)				Intermediate-Low-Risk PE 49 (41.9%)				Intermediate-High-Risk PE 20 (17.1%)				High-Risk PE 13 (11.1%)				*p*-Value
	Min	Mean ± SD		Max	Min	Mean ± SD		Max	Min	Mean ± SD		Max	Min	Mean ± SD		Max	
Age (years)	24	54.5 ± 14.2		81	41	65.7 ± 12.1		88	18	66.5 ± 16.3		91	46	68.3 ± 9.9		79	*0.001*
Gender (Male, Female)	12 (40%)		18 (60%)		33 (67.3%)		16 (32.7%)		11 (55%)		9 (45%)		6 (46.2%)		7 (53.8%)		0.104
BMI (kg/m^2^)	20.7	27.4 ± 4.9		40.4	17.1	26.5 ± 4.1		36.7	19.5	24.9 ± 3.5		30.8	21.1	25.3 ± 3.5		34.6	0.16
Dyspnea (No, %)	26 (86.7%)				45 (91.8%)				18 (90%)				13 (100%)				0.56
Syncope (No, %)	3 (10%)				3 (6.1%)				4 (20%)				2 (15.4%)				0.363
Chest pain (No, %)	9 (30%)				17 (34.7%)				6 (30%)				5 (38.5%)				0.93
Hemoptysis (No, %)	4 (13.3%)				8 (16.3%)				3 (15%)				4 (30.8%)				0.55
Smoking (No, %)	11 (36.7%)				19 (38.8%)				4 (20%)				5 (38.5%)				0.462
Hypertension (No, %)	14 (46.7%)				30 (61.2%)				7 (35%)				8 (61.5%)				0.185
Diabetes mellitus (No, %)	4 (13.3%)				9 (18.4%)				5 (25%)				1 (7.7%)				0.542
Deep vein thrombosis	15 (50%)				18 (36.7%)				4 (20%)				4 (33.3%)				0.18
Systolic blood pressure (mmHg)	105	130.1 ± 15.9		170	100	130.7 ± 18.7		200	90	115.75 ± 17.3		160	50	89.1 ± 15.6		125	*<0.001*
Dyastolic blood pressure (mmHg)	60	78.5 ± 11.1		100	60	80.2 ± 11.9		100	60	69.7 ± 10.1		90	20	56.3 ± 12.9		80	*<0.001*
Heart rate (bpm)	50	86.4 ± 23.8		130	42	94.4 ± 20.2		150	70	98.7 ± 25.8		150	30	103.3 ± 25.5		123	0.103
Oxygen saturation in ambient air (%)	89	95 ± 2.8		99	83	91.5 ± 3.8		98	81	90 ± 4.3		99	67	87.5 ± 6.4		95	*<0.001*
Wells score	0	4.2 ± 2.4		10.5	0	4 ± 2.3		9	0	3.5 ± 2.1		7.5	1.5	4.3 ± 1.1		6	0.08
Geneva score	2	8.1 ± 3.8		15	1	6.6 ± 3.2		16	0	6.2 ± 2.8		12	4	6.1 ± 1.2		9	0.67
Adverse cardiac events (No, %)	0 (0%)				7 (14.3%)				9 (45%)				10 (76.9%)				*<0.001*
Death within 30 days (No, %)	0 (0%)				2 (4.1%)				2 (10%)				5 (38.5%)				*<0.001*

BMI—body mass index. Italic style was used for statistically significant results.

**Table 3:** Biological and imagistic parameters of PE patients.

PE Group (No, %)	Low-Risk PE 30 (26.8%)			Intermediate-Low-Risk PE 49 (41.9%)			Intermediate-High-Risk PE 20 (17.1%)			High-Risk PE 13 (11.1%)			*p*-Value
	Min	Mean ± SD	Max	Min	Mean ± SD	Max	Min	Mean ± SD	Max	Min	Mean ± SD	Max	
Leucocytes (×10^9^/L)	5.83	10.66 ± 4.08	22.16	5.39	10.7 ± 4.26	23.78	4.06	9.78 ± 4.53	23.9	6.12	10.5 ± 3.38	23.9	0.862
Neutrophiles (×10^9^/L)	6.12	7.33 ± 3.69	17.84	2.30	8.03 ± 4.05	20.54	2.91	7.32 ± 4.18	20.45	4.05	8.06 ± 3.53	15.77	0.821
Hemoglobin (g/L)	9.2	13.1 ±1.7	17.6	9.3	12.9 ± 1.7	15.9	9.9	13.9 ± 1.9	17	9.1	12.8 ± 2.3	17.9	0.223
Thrombocytes (×10^9^/L)	76	252.37 ± 89.02	420	46	236.25 ± 105.47	420	119	225.95 ± 97.81	564	120	246.9 ± 199.1	745	0.213
CRP (mg/dL)	0.11	5. 59 ± 7.05	32.8	0.14	8.93 ± 18.14	118	0.08	5.76 ± 7.22	41.2	0.08	10.54 ± 11.48	118	0.550
Blood iron	12	61.52 ± 40.08	182	13	51.19 ± 28.48	131	21	55 ± 26.12	117	15	46.62 ± 23.28	105	0.538
Ferritin	67	390.9 ± 644.5	3085	43	442.78 ± 625.5	3085	114	430.94 ± 480.68	1986	123	419.82 ± 420.22	1633	0.991
LVEF (%)	45	54.77 ± 6.01	65	20	50.45 ± 8.01	62	48	52.1 ± 4.45	65	15	49.08 ± 10.89	60	0.061
D-dimer (µg/mL)	1.54	4.47 ± 1.35	6.1	1.19	4.64 ± 1.22	7.12	3.93	5.09 ± 0.61	6.2	2.07	5.26 ± 1.79	8.1	0.154
RVd (mm)	24	31.27 ± 5.31	46	28	36.14 ± 6.07	50	27	37.85 ± 5.50	50	25	39.15 ± 7.54	55	*0.002*
TAPSE (mm)	15	21.5 ± 2.92	26	12	18.58 ± 3.5	30	11	15.33 ± 2.95	24	12	14.54 ± 2.57	22	*<0.001*
sPAP (mmHg)	15	29.57 ± 11.13	50	15	37.69 ± 11.69	70	15	46.24 ± 12.54	68	16	55.08 ± 13.05	75	*<0.001*
RV/LV > 1 (No, %)	4 (11%)			17 (35.4%)			10 (47.6%)			10 (76.9%)			*<0.001*
Mastora score (points)	11	41.93 ± 21.03	84	13	59.63±24.05	107	42	83.57±17.34	126	51	101.23 ± 20.62	125	*<0.001*
	**Median (IQR)**			**Median (IQR)**			**Median (IQR)**			**Median (IQR)**			
hsTnI (ng/L)	22 (2.54–42.2)			26 (15–92.25)			112 (30.1–185.5)			101 (60–346)			*0.003*
NT-proBNP (pg/mL)	442 (200–662)			1377.5 (647–3334.25)			2488 (1615.5–3886)			5265 (4233–8328)			*<0.001*
Copeptin (pmol/L)	9.86 (6.89–17.30)			18.63 (9.31–35.76)			37.89 (25.04–51.01)			51.45 (36.5–75.09)			*<0.001*

LVEF—left ventricular ejection fraction, RVd—right ventricular diameter, TAPSE—tricuspid annular plane systolic elevation, sPAP—systolic pulmonary artery pressure, RV—right ventricle, LV—left ventricle, hsTnI—high sensitive troponin, NT-proBNP—N-terminal pro b-type natriuretic peptide. Italic style was used for statistically significant results.

**Table 4:** Correlation between copeptin and Mastora score and clinical, biologic and imagistic parameters.

Parameter	Copeptin		Mastora Score	
	r	*p*-Value	r	*p*-Value
Age	0.101	0.289	0.193	0.61
Sex	0.032	0.74	0.039	0.680
Diabetes mellitus	0.058	0.583	0.084	0.376
Arterial hypertension	0.057	0.549	0.063	0.511
Cancer	0.11	0.910	−0.011	0.907
BMI	−0.79	0.41	−0.64	0.50
SBP	−0.25	*0.006*	−0.282	*0.003*
HR	0.332	*0.001*	0.237	0.12
Oxygen saturation	−0.338	*<0.001*	−0.382	*0.001*
Hemoglobin	0.064	0.504	0.19	0.06
Leukocytes	0.137	0.15	0.04	0.675
Thrombocytes	−0.34	0.70	−0.65	0.5
CRP	0.235	*0.013*	0.030	0.757
RVd	0.312	*0.001*	0.416	*0.04*
TAPSE	−0.48	*0.001*	−0.473	*<0.001*
sPAP	0.523	0.072	0.639	0.064
RV/LV ratio > 1	0.442	*<0.001*	0.282	*0.03*
LVEF	−0.015	0.877	−0.079	0.409
hs cTnI	0.484	*<0.001*	0.324	*0.056*
D-dimers	0.326	*<0.001*	0.341	*0.01*
NT-proBNP	0.461	*<0.001*	0.37	*0.04*
Copeptin	1	0	0.535	*0.011*
Mastora score	0.535	*0.011*	1	0
Adverse events	0.467	*0.001*	0.542	*0.001*
Death	0.361	*<0.001*	0.287	*0.002*

BMI—body mass index, SBP—systolic blood pressure, HR—heart rate, CRP—C reactive protein, LVEF—left ventricular ejection fraction, RVd—right ventricular diameter, TAPSE—tricuspid annular plane systolic elevation, sPAP—systolic pulmonary artery pressure, RV—right ventricle, LV—left ventricle, hsTnI—highly sensitive troponin, NT-proBNP N-terminal pro b-type natriuretic peptide. Italic style was used for statistically significant results.

**Table 5:** AUC derived from ROC analysis for prediction of adverse events and death.

Parameter	Adverse Events			Death		
	Area Under Curve (AUC)	*p*-Value	95% Confidence Interval	Area Under Curve (AUC)	*p*-Value	95% Confidence Interval
Mastora score	0.871	<0.001	0.796–0.945	0.804	0.003	0.653–0.955
Copeptin	0.820	<0.001	0.725–0.914	0.883	<0.001	0.809–0.957
Copeptin + Mastora score	0.902	<0.001	0.841–0.962	0.850	0.001	0.724–0.967
Nt-proBNP	0.853	<0.001	0.783–0.922	0.855	<0.001	0.739–0.971
hsTnI	0.736	0.002	0.629–0.843	0.797	0.003	0.644–0.949
PESI score	0.742	0.001	0.649–0.834	0.810	0.002	0.688–0.932

hsTnI—highly sensitive troponin, NT—proBNP N-terminal pro b-type natriuretic peptide.

**Table 6:** Binary logistic regression analysis for adverse events and death in the PE group.

Biomarker	Adverse Events			Death		
	Exp B (Odds Ratio)	95% CI for Exp B	*p*-Value	Exp B (Odds Ratio)	95% CI for Exp B	*p*-Value
Copeptin	1.049	1.009–1.090	*0.015*	1.048	1.004–1.094	*0.032*
Mastora score	1.065	1.026–1.105	*0.001*	1.016	0.979–1.055	0.396
hsTnI	0.999	0.993–1.004	0.568	1.002	0.998–1.005	0.364
NT-proBNP	1.023	1.002–1.075	*0.048*	1.001	0.997–1.025	0.593
PESI score	0.997	0.974–1.021	0.818	1.024	0.995–1.054	0.102

hsTnI—highly sensitive troponin, NT-proBNP—N-terminal pro b-type natriuretic peptide. Italic style was used for statistically significant results.

## Data Availability

The data presented in this study are available within the article.
